# Amitriptyline inhibits bronchoconstriction independent of direct receptor binding and reduces number of caveolae

**DOI:** 10.1038/s41598-025-16935-0

**Published:** 2025-09-12

**Authors:** Virag Klein, Anna Michely, Paulina Hempel, Istvan Katona, Klaus Tenbrock, Christian Martin, Eva Verjans

**Affiliations:** 1 Department of Pediatrics, Medical Faculty, RWTH Aachen, University Hospital Aachen, Aachen, Germany; 2 Institute of Pharmacology and Toxicology, Medical Faculty, RWTH Aachen, University Hospital Aachen, Aachen, Germany; 3 Institute of Neuropathology, Medical Faculty, RWTH Aachen, University Hospital Aachen, Aachen, Germany; 4 Department of Neurology, The Houston Methodist Research Institute, Houston, TX USA

**Keywords:** Amitriptyline, Bronchoconstriction, Lipid rafts, Mechanism, Bronchial asthma, Pharmacology, Asthma

## Abstract

Bronchial asthma is a chronic inflammatory disease with rising prevalence worldwide. Apart from the immunological role of the tricyclic antidepressant amitriptyline in bronchial asthma, there is emerging evidence that inhaled amitriptyline directly reduces acute bronchoconstriction. However, the mechanism by which amitriptyline influences bronchial tone remains poorly understood. To influence bronchoconstriction, rat precision-cut lung slices treated with varying concentrations of amitriptyline (0–5 µM) and incubated with inhibitors targeting different signaling pathways. Amitriptyline reduces acetylcholine- and serotonin-induced bronchoconstriction. Neither the muscarinic antagonist ipratropium nor the phospholipase C inhibitor U73122, nor the protein kinase C inhibitor chelerythrine diminished the effect of amitriptyline. Inhibition of calcium sensitizing and induction failed to alter amitriptyline’s effect on bronchoconstriction. Caveolae—as part of the plasma membrane—display a microenvironment, where regulation of signal transduction takes place. Similar to methyl ß cyclodextrin (MBCD), a common substance to destroy caveolae, amitriptyline dramatically reduced the number of caveolae in lung tissue. However, unlike MBCD, this effect could not be explained by cholesterol depletion alone, as cholesterol repletion did not reverse amitriptyline’s effect. Furthermore, neither simvastatin (a lipid lowering agent) nor cytochalasin D (an inhibitor of actin polymerization), influenced the inhibitory effect of amitriptyline on bronchoconstriction. In conclusion, amitriptyline inhibits bronchoconstriction independently of direct receptor binding or interaction. It also reduces the total number of caveolae without effects on cholesterol lowering pathways or actin depolymerization. A more general mechanism seems likely, as inhibition of single signal transduction pathways failed. Further studies are required to elucidate the underlying mechanisms.

## Introduction

Bronchial asthma is a chronic inflammatory airway disease with increasing prevalence worldwide (up to 300 million people), in which first symptoms often arise in childhood^1,2^. One of the main characteristics is the acute bronchoconstriction occurring in asthma attacks. Airway smooth muscle cell contraction causes acute airflow obstruction, resulting in shortness of breath and wheezing^3^.

In a series of studies, we demonstrated that inhalation of amitriptyline, an inhibitor of acid sphingomyelinase and known as a tricyclic antidepressant^4^, directly influences acute bronchoconstriction in asthma supplementary to its immunomodulatory, anti-inflammatory effect^5,6^. However, the mechanism by which amitriptyline inhibits acute airway contraction and enhances airway dilatation is still unknown.

**Fig. 1:**
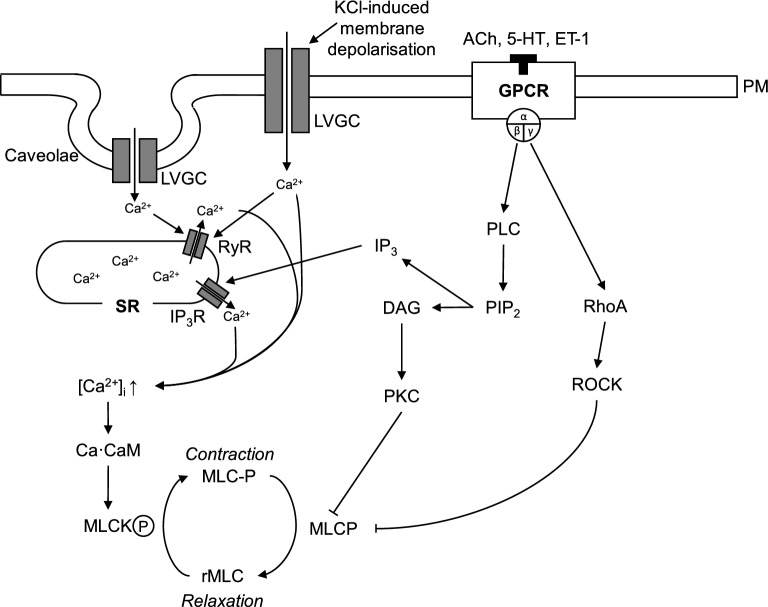
Illustration of signaling pathways leading to airway smooth muscle contraction. Classical pathways via PLC-PKC and RhoA/ROCK together with alternative pathways via different calcium channels in the plasma membrane and caveolae. ACh, acetylcholine; 5-HT, 5-hydroxytryptamine; ET-1, endothelin; PM, plasma membrane; DAG, diacylglycerol; GPCR, G protein-coupled receptors; IP3, inositol trisphosphate; IP3R, IP3 receptor; LVGC, L-type voltage-gated Ca2 + channel; MLCK, myosin light chain kinase; MLCP, myosin light chain phosphatase; PIP2, phosphatidylinositol 4,5-bisphosphate; PKC, protein kinase C; PLC, phospholipase C; rMLC, regulatory myosin light chain; ROCK, Rho kinase; RyR, ryanodine receptor; SR, sarcoplasmic reticulum.

Mechanistically, the binding of agonists (e.g. acetylcholine) to G-protein-coupled receptors, such as the M3-receptor, leads to activation of the phospholipase-C (PLC) signaling pathway (Fig. 1). From radioligand-studies, it is well-known that amitriptyline can bind to the M3 and other receptors of airway contraction, but the effect of this binding has not yet been studied^7^. Following the signaling pathway, PLC-mediated hydrolysis of membrane phospholipids results in the production of diacylglycerol (DAG) as well as inositol phosphates (IP3) and other choline metabolites. Inositol triphosphate increases calcium levels, while diacylglycerol activates protein kinase C (PKC), which then inhibits the myosin light chain phosphatase (MLCP)^8^. In the alternative pathway, receptor binding causes the activation of Rho and Rho kinase (ROCK), which again inhibits MLCP^9,10^. The findings of Matsunaga et al. point to a possible role of amitriptyline in the inhibition of phosphatidylinositol, the Ca^2^^+^-calmodulin-myosin light chain pathway and also possibly in the Rho-kinase pathway, but it proved difficult to define a single target of amitriptyline^11^.

Additionally, an increase in cytoplasmic Ca^2^^+^ concentration, caused by direct Ca^2^^+^ influx from the extracellular space, leads to contraction of airway smooth muscle cells. Upon depolarization of the cell membrane, voltage-gated Ca^2+^ channels open, allowing Ca^2+^ to flow into the cytoplasm^12^. Additionally, there are ligand-gated Ca^2+^ channels that open upon binding of agonists and activate the influx of Ca^2+^ ^13^. To some extent, this influx of Ca^2+^ is responsible for the opening of ryanodine receptors, which release additional Ca^2+^ from the sarcoplasmic reticulum into the cytoplasm^13^. In response to the overall rise in intracellular Ca^2^^+^, a complex with four Ca^2+^ ions and calmodulin, known as the calcium-calmodulin complex, is formed. This complex activates the myosin light chain kinase (MLCK) and leads to an increase in smooth muscle tone through phosphorylation of the regulatory light chain^14,15^. Further studies failed to show any involvement of amitriptyline in this process.

Several previous studies reported evidence for a role of lipid rafts and caveolae, a special type of lipid raft, in regulating Ca^2+^ homeostasis leading to smooth muscle contraction^16^. Caveolae are flask-shaped invaginations of the plasma membrane of different cell types, with a particularly high density in airway smooth muscle cells. Enriched in cholesterol, sphingolipids, and proteins, they are important in the G-protein coupled regulation of signaling molecules and intracellular signaling pathways^16–18^, including α-subunits of trimeric G proteins, PLC, the monomeric G protein RhoA and its downstream effector Rho kinase, and Ca^2^^+^-sensitive PKC isoforms^19–21^. A few studies linked the acid sphingomyelinase (ASM) to caveolae organization and stabilization^22^. By acting as an ASM-inhibitor, amitriptyline could perhaps influence the number, structure and/or function of caveolae.

In our study, we have investigated the mode of action of amitriptyline in acute bronchoconstriction. Clarification of the mechanism could offer a new therapeutic option, with inhaled amitriptyline playing a combined immunological and non-immunological role in asthmatic disease. Furthermore, the results of such studies could uncover new cellular targets for upcoming medication in bronchial asthma.

## Material and methods

### Animals and human lung biopsies

Experiments were performed on female 6–10 weeks old Wistar rats obtained from Janvier Labs (Le Genest-Saint-Isle, France). The study was approved by the regional governmental authorities and animal procedures were performed according to the German animal protection law (AZ 81–02.04.2018.A095, A4-30332A4) and is in accordance with ARRIVE guidelines.

### Precision-cut lung slices

Precision-cut lung slices (PCLS) were prepared as described before^23,24^. Briefly, intraperitoneal anesthesia in rats was performed with pentobarbital (Narcoren, Garbsen, Germany), and the adequate depth of anesthesia was confirmed by the absence of reflexes and cardiac activity. Thereafter, the trachea of rats was cannulated, and the abdomen and diaphragm opened up. The lungs were filled with warm 1.5% low-melting point agarose and cooled on ice. Tissue cores were prepared with a centered airway perpendicular to the long axis and cut into 250–300 µm thick slices with a Krumdieck tissue slicer (Alabama, Munford, AL, USA). PCLS were incubated at 37 °C with multiple medium changes conducted to remove residual agarose. Only slices free of agarose in a relaxed state, with beating cilia and an intact smooth muscle layer were used for the subsequent experiments 24-48 h hours after preparation.

### Concentration response curves with different mediators of bronchoconstriction, regulators of cholesterol pathways and pretreatments

To investigate the mode of action of amitriptyline on bronchoconstriction, PCLS of rat lungs were preincubated for 30 min with 0 µM, 0.1 µM, 1 µM and 5 µM amitriptyline dissolved in phosphate-buffered saline (total 1000µL). PCLS were additionally pre-treated for 30 min with ipratropium bromide, U73122, chelerythrine, Y-27632, MBCD, cholesterol, cytochalasin D for 60 min and simvastatin for 24 h or 72 h (total respective volume 1 ml). The PCLS were then treated with mediators of airway constriction (acetylcholine (10^–9^ – 10^–2^ M) and serotonin (10^–9^ – 10^–2^ M)) to calculate concentration response curves.

The following table shows the mediators used, their applied concentrations, targets, potential off-target effects, and the corresponding references used for dose determination and relevant (off-target) effects (Table 1):

**Table 1:** Mediators used, their applied concentrations, targtes, and potential off-targets used for dose determination and relevant effects.

Substance	Used concentration	Target	Off-target effects	Literature
Ipratropium bromide	1 nM	selective inhibitor of the M3 receptor	inhibition of (M1) receptors, anticholinergic effects, cholinergic effect by M2	^25^
U73122	10 µM	selective inhibitor of phospholipase C	Inhibition of phospholipase A2, effects on calcium channels, inhibition of 5-lipoxygenase	^26,27^
chelerythrine	10 µM	selective inhibitor of phosphokinase C	potential non-selective effect on other kinases at higher dosage, inhibits the BclXL-Bak BH3	^28,29^
Y-27632	10 µM	selective inhibitor of rho-kinase	potential non-selective effect on other kinases at higher dosage, inhibitor of PKC epsilon and PKN2	^30,31^
MBCD	3.2 mM	removes cholesterol from lipid raft domains	Carrier for lipophilic drugs, actin depolymerization	^12,32–34^
cholesterol	6.5 µM	central component of lipid rafts	Membrane rigidity and reduced fluidity	^35^
cytochalasin D	10 µM	actin polymerization inhibitor	alters membrane trafficking, mitochondrial dysfunction, disruption of formation of actin polymers and activates p53	^36–38^
simvastatin	10 µM	competitive inhibitor of the HMG-CoA reductase, cholesterol lowering effect	mitochondrial dysfunction, inhibition of protein prenylation, MEK, ERK and p38 inhibition	^39,40^

During the experiment, the intraluminal area of the airways was monitored with a digital video camera (USB cameras, Supervision). For measurement of bronchoconstriction, PCLS were placed into a culture dish and were fixed by a platinum wire with threads to the bottom of the dish to avoid movement during measurement. Images were recorded every 5 s for a time period of 5 min until the next concentration was added. The images were analyzed with Optimas 6.5 (Media Cybernetics, Bothell, WA) and ImageJ software. The airway area before addition of the lowest concentration of the agonist was defined as 100%. Bronchoconstriction was expressed as a percentage of the initial airway area (IAA). GraphPad Prism 9 software (GraphPad Software, San Diego, CA, USA) was used for fitting sigmoidal concentration response curves.

### Ca^2+^-dependent signaling pathways

To determine amitriptyline´s influence on Ca^2^^+^-dependent signaling pathways, PCLS from rats were preincubated with 0 µM, 0.1 µM, 1 µM, and 5 µM amitriptyline for 30 min suspended in phosphate-buffered saline solution (total volume 1000 µl) and 100 mM KCl. Potassium chloride acts as a stimulus to bypass G protein-coupled receptors (GPCR) and activate smooth muscle by direct membrane depolarisation, thereby inducing bronchoconstriction. To assess the influence of amitriptyline on voltage-gated Ca^2+^-channels, PCLS were additionally preincubated with 10 µM nifedipine for 30 min and bronchoconstriction was induced by serotonin. Images were captured and analyzed as described above.

### Transmission electron microscopy

To examine the caveolae of airway smooth muscle cells, PCLS of rats were processed as described above. To further prepare for transmission electron microscopy, ultrathin sections from glutaraldehyde-fixed and epoxy resin-embedded PCLS were mounted on grids^41,42^, images were obtained by electron microscopy using a Zeiss EM900 transmission electron microscope with the magnification of 30000X at 80 kV acceleration voltage.

To assess the number of intact caveolae, the following criteria were defined: omega- or flask-shaped invaginations of the plasma membrane, clearly marked black outline, with a hollow interior. Horizontally and vertically cut caveolae were counted; flattened caveolae and caveolae that were reduced in dimension were not. Using these criteria, three independent experts quantified the numbers of caveolae in all images, using the DotDotGoose software version 1.7.0 (American Museum of Natural History; Center for Biodiversity and Conservation; New York). All images were blinded for names and treatment. The median of all counts belonging to each picture was used for further evaluation.

### Statistical analysis

Quantitative results are presented as means ± standard error of the mean (SEM). The data were analyzed for normal distribution using the Shapiro–Wilk test. Homoscedasticity was tested using the covtest statement. In case of heteroscedasticity of data, degrees of freedom were adjusted using the Kenward-Roger approximation. Statistical analyses of concentration–response curves were conducted using Schild regression analysis (GraphPad Prism 5.0). To analyze for significant effects, EC_50_ values of the Schild fitted concentration–response curves were analyzed by one-way ANOVA, with Tukey’s correction for multi-comparison. Due to the fact that we did observe a significant difference between the control group and the group treated with amitriptyline, we decided to focus on evaluating only the effects compared to amitriptyline using a one-way ANOVA to specifically and more accurately address the research question. Figure 5B is the only figure in which different treatments were statistically compared with each other and with amitriptyline using a two-way ANOVA, with Tukey’s correction for multi-comparison.

Here, we were interested in comparisons between different treatments groups and between treatment groups and controls. To analyze kinetic curves (Fig. 3 D-G) for significant effects, means of the stabilized end-states were analyzed by one-way ANOVA, with Tukey’s correction for multi-comparison. A p-value of < 0.05 was considered statistically significant (* *p* < 0.05, ** *p* < 0.01, *** *p* < 0.001).

## Results

Other studies conducted by our group showed that the antidepressant amitriptyline inhibits acetylcholine-, histamine- and serotonin-induced airway contraction, but the underlying mechanism is unclear. Here we show an illustration of known signaling pathways leading to airway smooth muscle contraction (Fig. 1). All components of these signaling pathways are possible targets of amitriptyline binding or interaction.

**Fig. 2:**
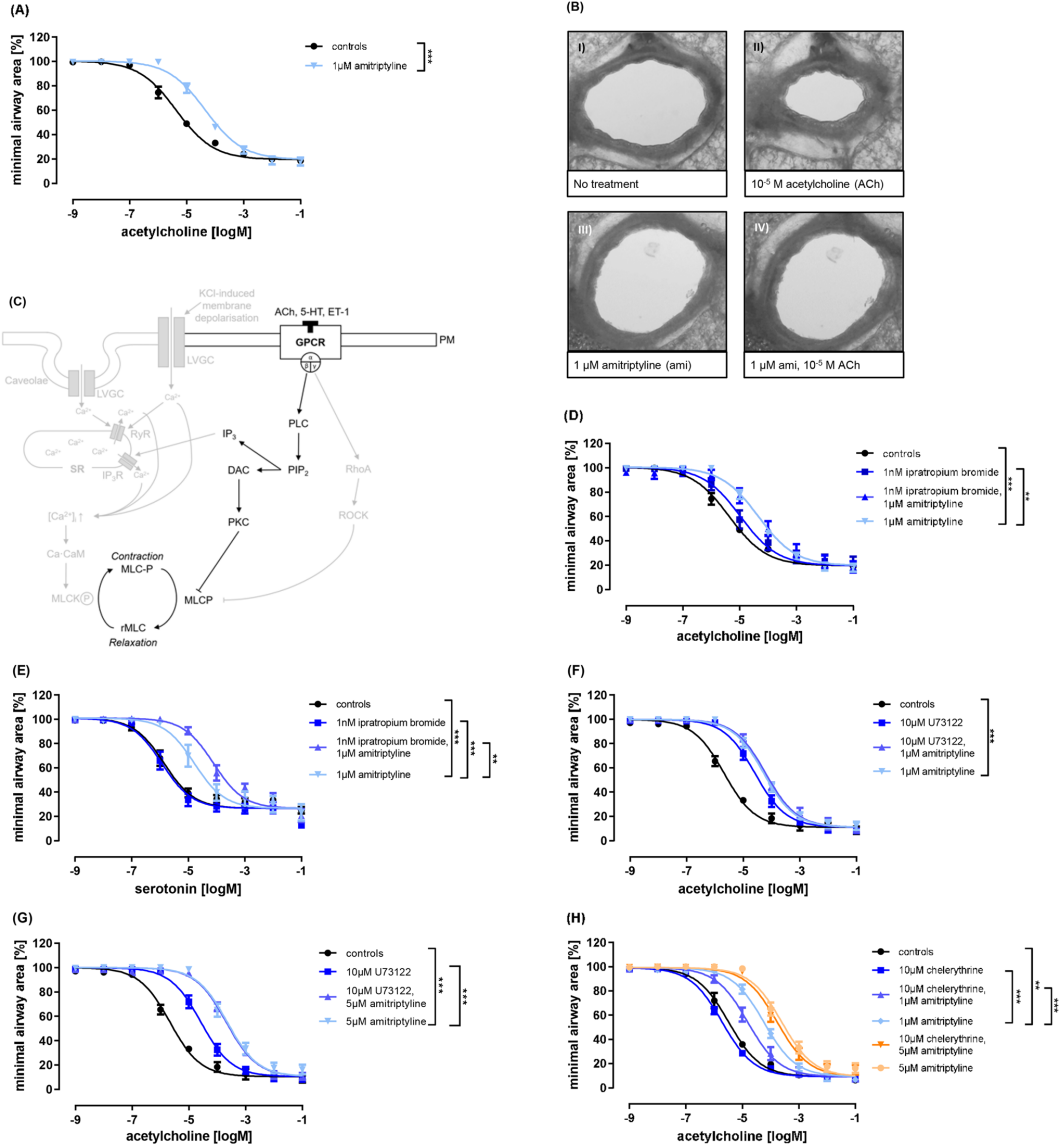
Amitriptyline inhibits acetylcholine- and serotonin-mediated airway contraction beyond PLC/PKC inhibition. (**A**) Concentration–response curve of rat PCLS stimulated with acetylcholine and pretreated with amitriptyline (1 µM). (**B**) Video-microscopic images of rats from (**A**). (**C**) Illustration of the part of the signal transduction cascade studied in Fig. 2. **(D-H)** Concentration–response curves of rat PCLS stimulated with acetylcholine (**D**, **F**–**H**) or serotonin (**E**) and pretreated with ipratropium bromide (1 µM) (**D**,**E**), U73122 (10 µM) (**F**,**G**) or chelerythrine (10 µM) **(H)** and amitriptyline (1 and 5 µM) or combinations. All graphs represent means ± SEM; *p ≤ 0.05, ** p ≤ 0.01, *** p ≤ 0.001. n = 4–5 in all groups. Up to three slices per condition were measured for each animal.

First, we used the model of precision-cut lung slices from rats, which were preincubated with 1 µM amitriptyline and afterward stimulated with acetylcholine in rising concentrations. The concentration–response curve of amitriptyline-treated slices showed a significant shift to the right as a sign of reduced airway contraction compared to PBS controls (Fig. 2 (A)). Microscopic images of PCLS with and without amitriptyline treatment are shown in Fig. 2 (B).

It has long been acknowledged that acetylcholine induces airway contraction by M3-receptor binding and activation of downstream signaling pathways via phospholipase C (PLC), protein kinase C (PKC), and inhibition of myosin light chain kinase (MLCP) (Fig. 2 (C)). Studies from the 1990s demonstrated a direct binding of amitriptyline to the M3 receptor. To determine the potential role of amitriptyline in this pathway, we first pre-incubated rat PCLS with ipratropium bromide, a competitive muscarinic receptor inhibitor, in combination with or without additional amitriptyline (Fig. 2 (D)). PCLS, preincubated with amitriptyline, showed strong inhibition of bronchoconstriction, illustrated by an additional right shift of the corresponding concentration–response curve after acetylcholine stimulation. The combination of pre-incubation with amitriptyline and ipratropium bromide did not result in any significant changes compared to the solely amitriptyline-treated slices, indicating that an inhibition/blockade of the M3 receptor cannot decrease or change the effect of amitriptyline.

Next, we performed serotonin-induced bronchoconstriction in rat PCLS. As expected, the M3-receptor inhibitor ipratropium bromide had no effect on 5-HT_A_-receptor-mediated pathways but amitriptyline-preincubation again resulted in a significant right shift of the corresponding concentration–response curve. Surprisingly, the combination of muscarinic receptor antagonist and amitriptyline led to stronger inhibition of airway contraction compared to amitriptyline alone (Fig. 2 (E)).

Following downstream signaling of G-protein coupled receptors like M3 and 5-HT2 we next focused on PLC and PKC inhibition during amitriptyline treatment. U-73122, as a selective inhibitor of PLC, led to the inhibition of airway contraction via acetylcholine. Treatment with amitriptyline in different non-toxic concentrations (1 µM and 5 µM) resulted in stronger inhibition of airway contraction compared to U-73122 alone, while the combination of both produced no additional effect (Fig. 2 (F-G)). Thus, the effect of amitriptyline seems to be independent of PLC inhibition. PKC inhibition via chelerythrine led to an inhibition of airway contraction but was again weaker than the effect of amitriptyline (Fig. 2 (H)). Surprisingly, the combination of a PKC inhibitor and amitriptyline led to concentration-dependent changes. The pre-incubation with 1 µM amitriptyline resulted in a weak left shift of the concentration–response curve, so PKC inhibition seemed to weaken the effect of amitriptyline. On the other hand, a higher concentration of amitriptyline with 5 µM led to stronger inhibition of bronchoconstriction. We cannot therefore conclude that amitriptyline works independently of PKC, but that PKC seems to be a less likely target.

**Fig. 3:**
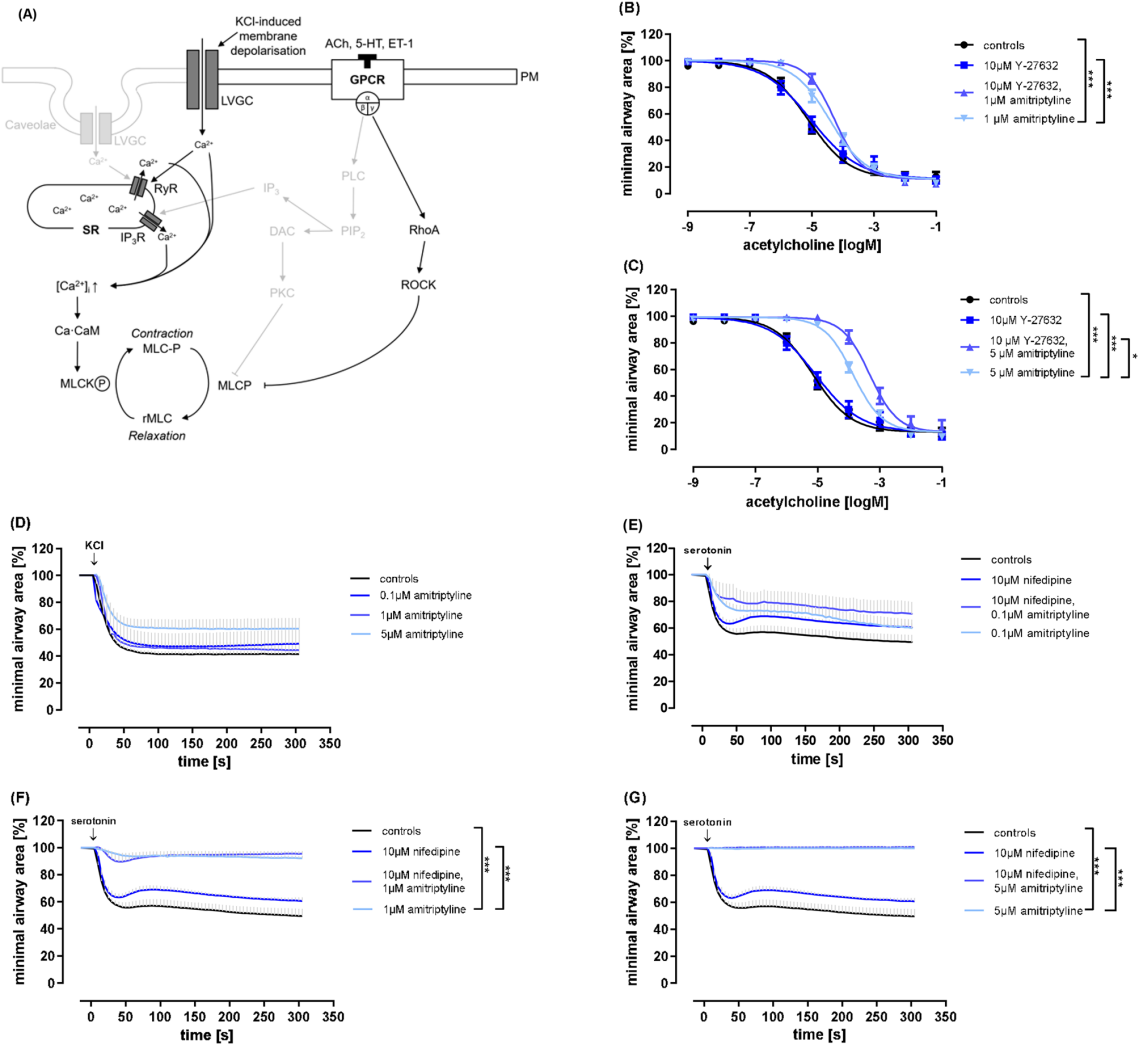
Amitriptyline does not directly inhibit alternative pathways of bronchoconstriction. (**A**) Illustration of signaling pathways studied. (**B**,**C**) Concentration–response curves of rat PCLS stimulated with acetylcholine and pretreated with Y-27632 (10 µM) and amitriptyline (1 or 5 µM) or combinations. (**D**-**G**) Kinetic curves of rat PCLS stimulated with KCl (**D**) or serotonin (**E**–**G**) and pre-incubated with amitriptyline (0,1 µM, 1 µM, 5 µM) (**D**), or its combination with nifedipine (10 µM) (**E**–**G**). All graphs represent means ± SEM; * p ≤ 0.05, *** p ≤ 0.001. n = 3 in all groups. Up to three slices per condition were measured for each animal.

Next, we focused on alternative pathways of smooth muscle cell contraction, both Ca^2^^+^-dependent and Ca^2+^-sensitizing pathways (Fig. 3 (A)). Treatment with 10 µM Y-27632, a selective RhoA/ROCK inhibitor, alone did not have any effect on acetylcholine-induced bronchoconstriction. While Y-27632 and 1 µM amitriptyline led to the same inhibition of airway contraction, pre-incubation with Y-27632 and 5 µM amitriptyline resulted in stronger inhibition of bronchoconstriction than treatment with amitriptyline alone (Fig. 3 (B-C)). Thus, it appears either that the effect of amitriptyline is independent of RhoA/ROCK inhibition or that alternative signaling pathways (e.g. PKC/PLC) are activated when RhoA/ROCK are blocked.

Several studies have demonstrated the direct influence of calcium influx on smooth muscle cell contraction through L-type voltage-gated calcium channels (LVGCs) and membrane depolarization^13,14^. Here, treatment with KCl is used to decrease the resting membrane potential and cause strong airway contraction. These effects can be suppressed with the selective L-type Ca^2+^ channel blocker nifedipine. Although treatment with amitriptyline alone in different non-toxic concentrations (0.1 µM, 1 µM, and 5 µM) failed to cause significant changes in airway contraction after addition of KCl, we observed a trend towards weaker contraction (Fig. 3 (D)). The activation of L-type voltage-gated calcium channels (LVGCs) allows the entry of calcium ions into the cell, leading to muscle contraction. Next, we induced contraction of airways in rat PCLS using 10^–5.5^ µM serotonin. Preincubation with 10 µM nifedipine, a typical LVGC-blocker, did not result in significant changes in bronchoconstriction. Amitriptyline inhibited airway contraction in a concentration-dependent manner, with complete inhibition at 5 µM (Fig. 3 (E–G)). The combination of nifedipine and amitriptyline did not strengthen these effects. Thus, the action of amitriptyline appears to be independent of an inhibition of L-type voltage-gated channels. In summary, we could not link amitriptyline´s mode of action to Rho-kinase or any alternative Ca^2+^ signaling pathways.

**Fig. 4:**
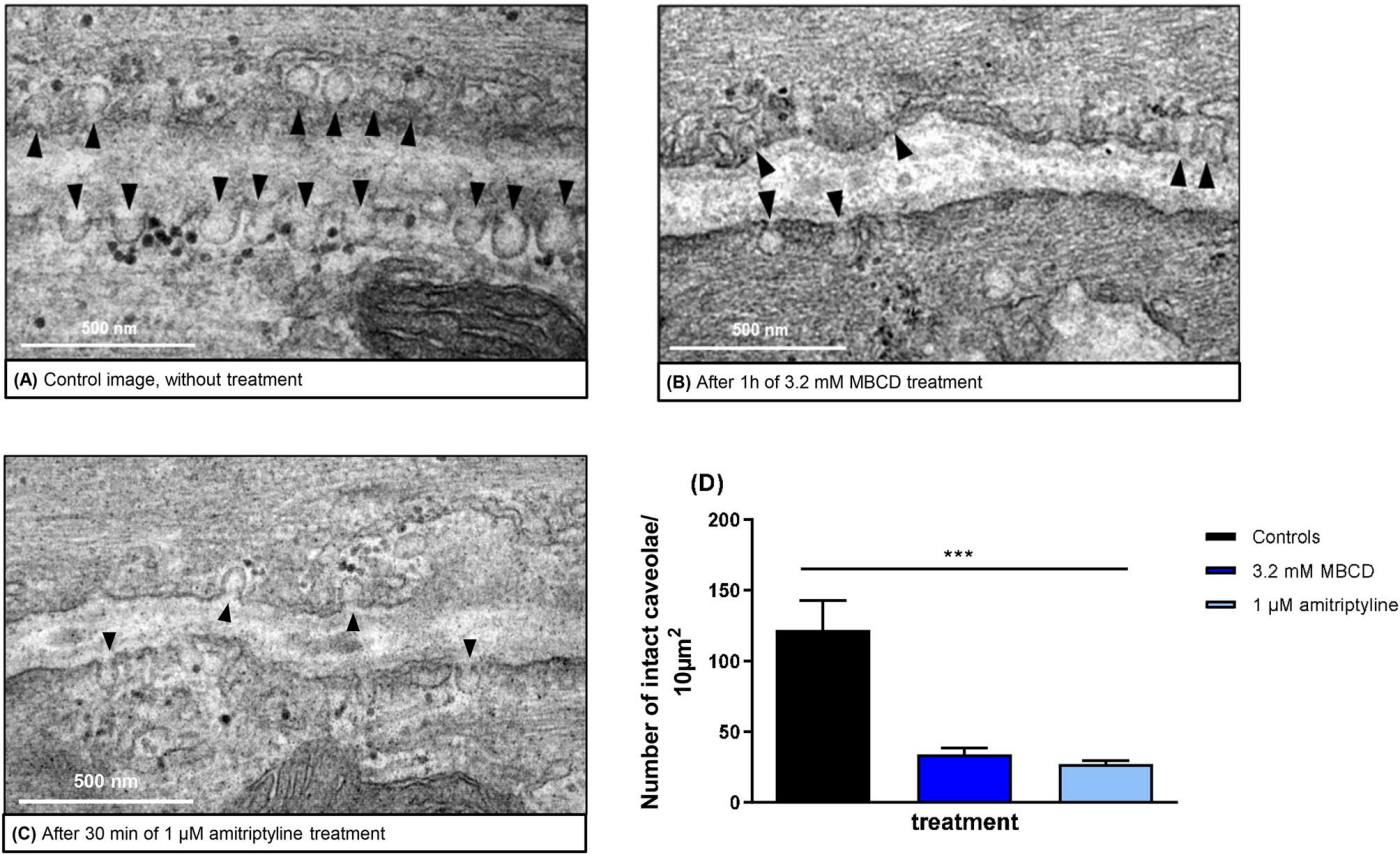
Amitriptyline reduces number of caveolae comparable to MBCD. (**A**-**C**) Transmission electron microscopy images of airway smooth muscle cells of rats derived from PCLS, without treatment (**A**), with MBCD (3.2 mM) (**B**) and amitriptyline (1 µM) (**C**). The black arrows point to typical caveolae. (**D**) Number of intact caveolae per 10µm^2^ after MBCD (3.2 mM) and amitriptyline (1 µM) treatment. All graphs represent means ± SEM; *** p ≤ 0.001. n = 3 in all groups. Up to three slices per condition were measured for each animal.

Membrane (lipid) rafts, and particularly caveolae, a specialized subset, are cellular domains that concentrate plasma membrane proteins such as signalling receptors and various lipids involved in regulating cell functions, including bronchoconstriction^43^. In this study, we investigated the effect of amitriptyline on caveolae as regions of importance for signalling transduction of airway contraction. Treatment of rat PCLS with amitriptyline for 30 min significantly reduced the number of caveolae in the plasma membrane (Fig. 4 (B) and (D)). Next, we used MBCD, a known cholesterol-depleting agent which induced a similarly strong disruption of caveolae in rat PCLS at 3.2 mM (Fig. 4(C-D)). At this concentration, MBCD showed no relevant toxicity in LDH assay (data not shown).

**Fig. 5:**
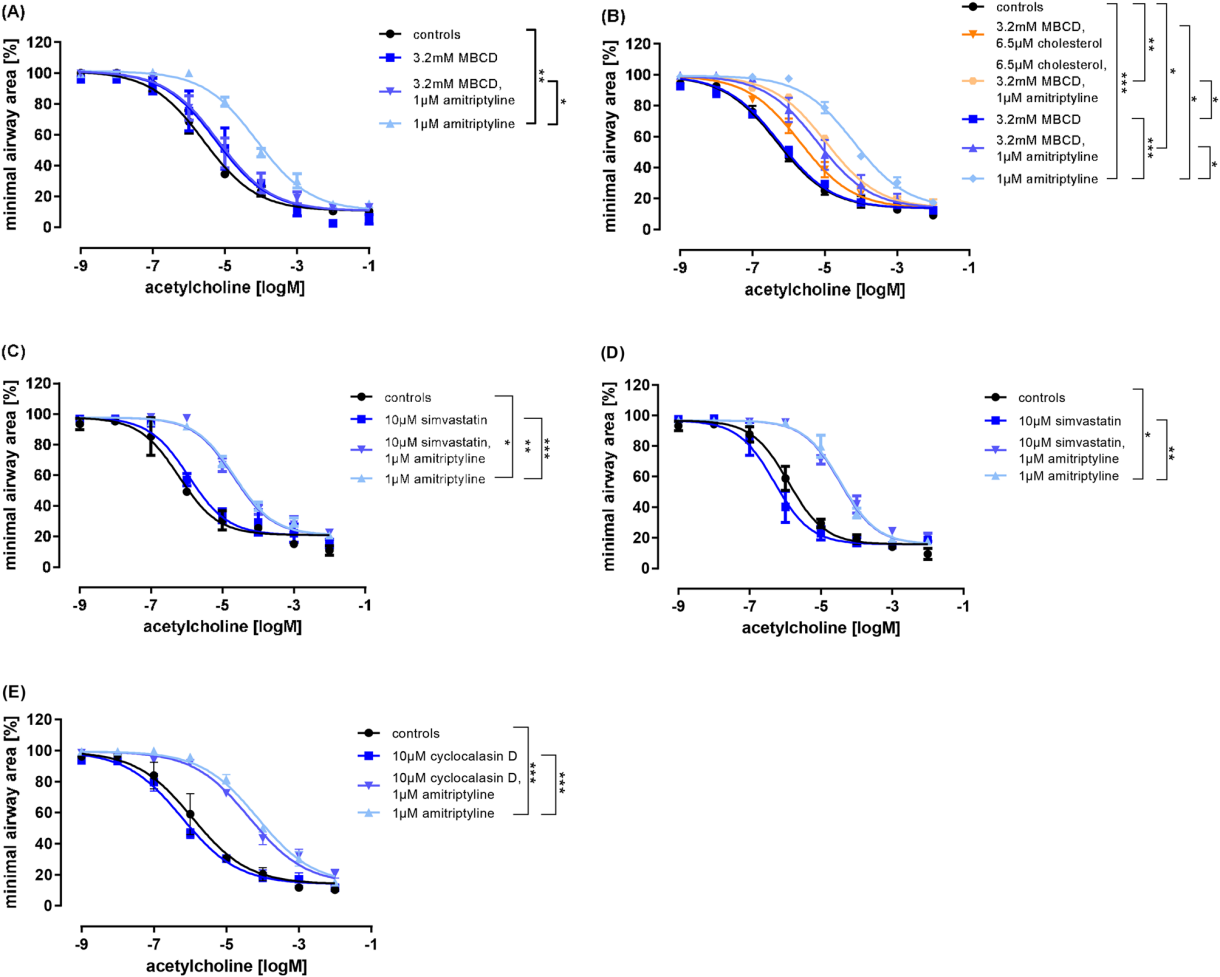
The inhibitory effect of amitriptyline on bronchoconstriction is independent of cholesterol-lowering mechanisms or actin (de)polymerization. (**A**–**E**) Concentration–response curves of rat PCLS stimulated with acetylcholine and pretreated with 1 µM amitriptyline and 3.2 mM MBCD (**A**), 3.2 mM MBCD, 6.5 µM cholesterol, 1 µM amitriptyline (**B**), 1 µm amitriptyline,10 µM simvastatin for 24 h (**C**) or 72 h (**D**) or 10 µM cyclocalasin D, 1 µM amitriptyline and combinations (**E**).

Next, we preincubated rat PCLS with MBCD, amitriptyline, or a combination of both, and then induced acetylcholine-dependent airway contraction. As expected, amitriptyline alone significantly reduced airway-contraction (Fig. 5 (A)). No significant differences were observed between MBCD alone and the control group. Interestingly, the combination of MBCD and amitriptyline reduced the inhibitory effect of amitriptyline on airway contraction.

It is well known, that MBCD depletes cholesterol from cellular membranes by forming inclusion complexes, thereby disrupting lipid rafts and affecting membrane-associated signalling pathways. Additionally, MBCD leads also to actin depolymerisation^44^.

To determine whether cholesterol repletion could restore amitriptyline’s effect under addition of MBCD, we treated rat PCLS with MBCD and cholesterol, either alone or in combination with amitriptyline (Fig. 5 (B)). Due to the poor solubility of pure cholesterol in aqueous solutions (< 24 nM), MBCD was used as a cholesterol carrier^33^. The combination of MBCD, amitriptyline and cholesterol did not reverse the effect of MBCD, but the effect of amitriptyline was still inhibited. Therefore, it is less likely, that the inhibitory effect of amitriptyline on bronchoconstriction is cholesterol-dependent.

To further prove, if cholesterol-lowering by decreasing cholesterol production contribute to the action of amitriptyline, we tested the inhibition of HMG CoA reductase to lower cholesterol levels. Rat PCLS were treated with simvastatin, a well-established lipid-lowering agent^39^, for either 24 h (Fig. 5 (C)) or 72 h (Fig. 5 (D)). The statin did not affect bronchoconstriction on its own. No significant differences were observed between slices treated with amitriptyline alone and those treated with a combination of amitriptyline and simvastatin. Therefore, we suggest that the effect of amitriptyline is not dependent on cholesterol reduction.

To investigate if actin (de)polymerisation is involved in amitriptyline’s effect, rat PCLS were pretreated with cytochalasin D (CD), an inhibitor of actin polymerization, for 1 h followed by stimulation with increasing concentrations of acetylcholine (Fig. 5 (E)). As expected, slices treated with amitriptyline alone showed a significant reduction in bronchoconstriction. However, the slices treated with the combination of amitriptyline and CD showed no significant difference compared to those treated with amitriptyline alone. This indicates that the effect of amitriptyline does not depend on actin (de)polymerization.

All graphs represent means ± SEM; *** p ≤ 0.001. n = 3 in all groups. Up to three slices per condition were measured for each animal.

In summary, amitriptyline inhibits bronchoconstriction independently of direct receptor binding or interaction and reduces the number of caveolae independently of cholesterol-lowering pathways and actin (de)polymerization.

## Discussion

Here we provide evidence that amitriptyline directly inhibits acute acetylcholine- and serotonin-induced airway contraction, one main features of asthma medication. The findings indicate that amitriptyline brings direct change neither in G-protein coupled receptor activation, nor in downstream signaling pathways of bronchoconstriction, nor in ion channel mediated calcium influx. Instead, it seems to operate in lipid rafts and caveolae, a raft subtype known to be cholesterol and caveolin-1-rich domains.

Amitriptyline, a tricyclic antidepressant, is known for its effects on the nervous system, primarily used in patients with depression^45–47^. It acts by blocking the reuptake of the neurotransmitter’s serotonin and norepinephrine, thus influencing mood regulation^48^. Despite its well-established role in treatment of depression, only a few studies have explored the role of amitriptyline in smooth muscle cell contraction or airway disease.

Years ago, the group of Dahlin and Ryrfeldt et al. demonstrated amitriptyline-induced acute vaso- and bronchoconstriction in isolated perfused and ventilated rat lungs. Amitriptyline exposure of 50 to100µM caused airway contraction as well as a decrease of the perfusion flow, both of which could be inhibited by the protein kinase inhibitor staurosporine and the endothelin A/B receptor antagonist PD 145,065^49,50^. In contrast, Matsunaga et al. reported that amitriptyline does not influence smooth muscle reactivity in isolated tracheal rings of rats at concentrations of < 1 µM. Higher amitriptyline dosage caused inhibition of tracheal contraction, partly through inhibition of phosphatidylinositol (PI) metabolism but also possibly through inhibition of the Rho-kinase pathway^11^. Hence, the effect of amitriptyline seems to be dose- and tissue-dependent. In previous studies in our lab, we conducted toxicity tests with amitriptyline and confirmed that the concentrations of amitriptyline used in our study, ranging between 0.1 µM and 5 µm, were non-toxic (results not shown), whereas higher dosages showed increasing toxicity, leading to cellular stress and cell death. Additionally, we confirmed the inhibitory effect on bronchoconstriction induced by both systemic and inhaled amitriptyline (≤ 5 µM). Moreover, it effectively dilated pre-contracted airways, displaying an efficacy comparable to typical bronchodilation with IBMX and/or ß2-sympathomimetics^5,51^. Amitriptyline could therefore be the first therapeutic agent in asthmatic disease to have strong effects on the T_H_2-allergic phenotype and on acute bronchoconstriction^6^.

But how does it work? How does amitriptyline inhibit airway contraction in acute asthma attacks? Receptor binding studies from the 1990s demonstrated a direct binding of amitriptyline to the muscarinic acetylcholine receptor and other receptors involved in airway contraction. But the effect of this interaction remained unclear. In our study, we showed that amitriptyline does not directly influence typical pathways of bronchoconstriction via blockade of PLC/PKC, Rho-Kinase or via different Ca^2+^-ion-channels. This we tested by blocking these pathways with specific inhibitors to see if amitriptyline interferes with signalling molecules or receptors. But, the effect of amitriptyline was the same when different parts of typical pathways of bronchoconstriction were blocked.

Caveolae are characterized by different cholesterol-binding proteins, the caveolins, that form an oligomeric network along the inner leaflet of the plasma membrane^52^. Hereby, caveolin-1 is the primary caveolin protein expressed by smooth muscle cells, also in airways.

Keshavarz et al. showed that caveolae abundancy was reduced in caveolin-1-knockout mice, while acetylcholine-induced airway contraction was maintained^53,54^. Treatment of PLCS from caveolin-1-knockout mice with MBCD reduced acetylcholine-induced constriction by about 50%, indicating that either intact caveolae with enough calveolin-1 or colocalization of caveolin-1 and muscarinic receptors play a key role in muscarine-induced bronchoconstriction. In line with these data, we demonstrated that amitriptyline strongly inhibited bronchoconstriction also when the M3-receptor was blocked and displayed an even stronger effect than typical blockade of the M3 receptor with ipratropiumbromide.

In our study, MBCD alone did not alter acetylcholine-induced airway contraction. This was in line with results of Gosens et al., who reported that treatment of airway smooth muscle cells with MBCD disrupted the colocalization of caveolin-1 and muscarinic M3-receptor in airway smooth muscle cells. But this this had no effect on muscarinic receptor availability or maximal airway contraction^17^. However, combined treatment of PCLS with MBCD and amitriptyline reduced the inhibitory effect of amitriptyline on acetylcholine-induced bronchoconstriction in our study. Since this effect could not be reproduced with simvastatin, a compound also known for its cholesterol-lowering properties, it is unlikely that amitriptyline’s mechanism of action relies on cholesterol depletion similar to MBCD. Beyond its role in disrupting caveolae, MBCD is also recognized as an actin depolymerizing agent, which increases plasma membrane permeability^44^. We treated PCLS with cytochalasin D, an established inhibitor of actin polymerization, and found no effect on amitriptyline’s inhibitory action, suggesting that actin remodeling is not essential for its function. Our experiments further showed that the bronchodilatory effect of amitriptyline remains supressed even after cholesterol repletion, when PCLS were treated with a combination of cholesterol, MBCD and amitriptyline. This is notable since cholesterol repletion typically mitigates the effects of MBCD by restoring membrane cholesterol levels and re-establishing lipid raft integrity^45^.

Despite all described mechanisms, MBCD is widely used as a solubilising agent for lipophilic drugs, including amitriptyline^32,44^. The fact that the inhibitory effect of amitriptyline on the bronchoconstriction is still supressed in the presence of MBCD, suggests that MBCD may bind amitriptyline non-covalently, reducing its effective concentration.

For the first time, we clearly demonstrated, that amitriptyline reduces the number of caveolae in bronchial tissue. We propose, that this effect is independent of the acid sphingomyelinase (ASM), as in previous studies we were able to show, that the constriction of bronchial airways by acetylcholine was not changed by an ASM-deficiency (Smpd1-/-)^5^.

Previous studies in the past demonstrated that different regulators of lipid rafts or caveolae were protective in bronchial asthma as anti-inflammatory and anti-obstructive medication as well. Intranasally administered Apolipoprotein A, which facilitates removal of excess cholesterol and leads to caveolae reorganization, had anti-inflammatory effects, possibly dependent on IL-33 inhibition and decreased expression of the epithelial mucus gene Muc5ac, an epithelial cytokine which can promote eosinophilic inflammation in the airways^55^. Additionally, AIBP (apolipoprotein A-I binding protein) interacted with phosphatidylinositol 3-phosphate, which was associated with a rearrangement of the actin cytoskeleton and modified downstream-signalling^56^.

We hypothesize that amitriptyline-induced reduction in the number of caveolae seems to inhibit bronchoconstriction, which could be associated with a lower number or a rearrangement of bronchoconstriction-associated receptors within these membrane microdomains.

In conclusion, amitriptyline appears to be a potent candidate for the treatment of bronchial asthma with strong anti-inflammatory and anti-obstructive effects^6^. Its mechanism of action seems to involve the disruption or reorganization of caveolae within lipid rafts. Further studies are required to elucidate the precise mechanism involved and to assess the therapeutic potential of amitriptyline in human respiratory diseases.

## Data Availability

The datasets supporting the conclusions of this article are included within the article.
